# Methyl β-Cyclodextrin-sperm-mediated gene editing (MBCD-SMGE): a simple and efficient method for targeted mutant mouse production

**DOI:** 10.1186/s12575-024-00230-9

**Published:** 2024-01-26

**Authors:** Parisa Moradbeigi, Sara Hosseini, Mohammad Salehi, Asghar Mogheiseh

**Affiliations:** 1 Department of Clinical Sciences, School of Veterinary Medicine, Shiraz University, P. O. Box: 7144169155, Shiraz, Iran; 2 Cellular and Molecular Biology Research Center, Shahid Beheshti University of Medical Sciences, P.O. Box: 193954717, Tehran, Iran; 3 Hasti Noavaran Gene Royan Co, Tehran, Iran; 4 Department of Biotechnology, School of Advanced Technologies in Medicine, Shahid Beheshti University of Medical Sciences, Tehran, Iran

**Keywords:** Sperm-mediated gene Transfer (SMGT), CRISPR/Cas9, Methyl β-cyclodextrin (MBCD), Targeted mutant mouse, Reactive oxygen species (ROS), Sperm-mediated gene editing (SMGE), and Gene uptake

## Abstract

**Background:**

Generating targeted mutant mice is a crucial technology in biomedical research. This study focuses on optimizing the CRISPR/Cas9 system uptake into sperm cells using the methyl β-cyclodextrin-sperm-mediated gene transfer (MBCD-SMGT) technique to generate targeted mutant blastocysts and mice efficiently. Additionally, the present study elucidates the roles of cholesterol and reactive oxygen species (ROS) in the exogenous DNA uptake by sperm.

**Results:**

In this study, B6D2F1 mouse sperm were incubated in the c-TYH medium with different concentrations of MBCD (0, 0.75, 1, and 2 mM) in the presence of 20 ng/µl pCAG-eCas9-GFP-U6-gRNA (pgRNA-Cas9) for 30 min. Functional parameters, extracellular ROS, and the copy numbers of internalized plasmid per sperm cell were evaluated. Subsequently, in vitro fertilization (IVF) was performed and fertilization rate, early embryonic development, and transfection rate were assessed. Finally, our study investigated the potential of the MBCD-SMGT technique in combination with the CRISPR-Cas9 system, referred to as MBCD-SMGE (MBCD-sperm-mediated gene editing), for generating targeted mutant blastocysts and mice. Results indicated that cholesterol removal from the sperm membrane using MBCD resulted in a premature acrosomal reaction, an increase in extracellular ROS levels, and a dose-dependent influence on the copy numbers of the internalized plasmids per sperm cell. Moreover, the MBCD-SMGT technique led to a larger population of transfected motile sperm and a higher production rate of GFP-positive blastocysts. Additionally, the current study validated the targeted indel in blastocyst and mouse derived from MBCD-SMGE technique.

**Conclusion:**

Overall, this study highlights the significant potential of the MBCD-SMGE technique for generating targeted mutant mice. It holds enormous promise for modeling human diseases and improving desirable traits in animals.

**Graphical Abstract:**

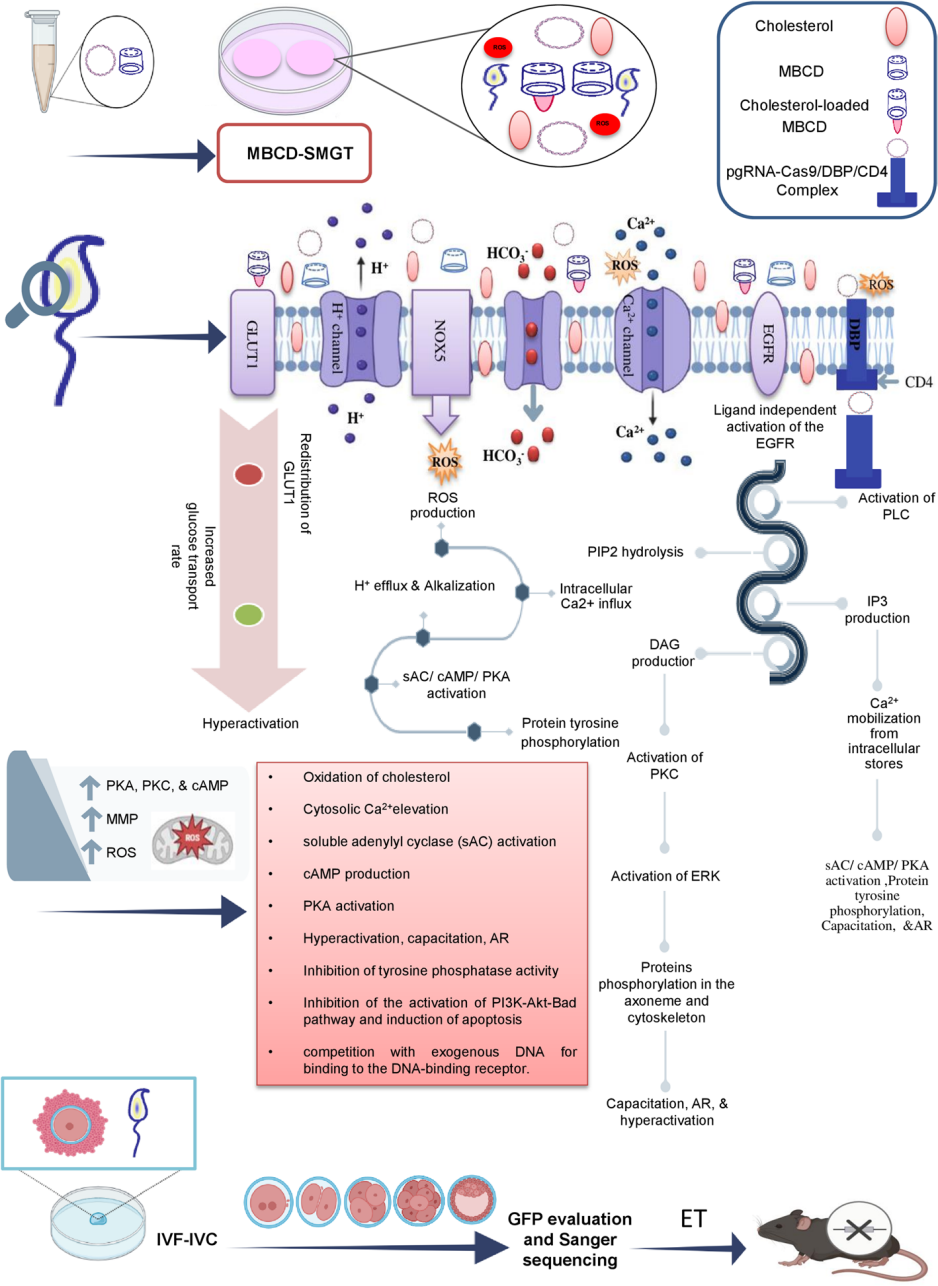

## Background

Targeted genetic modifications are utilized in scientific research to investigate gene function, associate mutations with physiological and pathological phenotypes, and improve desirable traits in animals. They also play a vital role in generating animal models for studying human diseases and developing novel therapies [1]. Previously, producing animal models has been a costly and intricate process involving embryonic stem cell manipulation and mouse breeding to acquire the desired phenotype and genotype. However, since the advent of clustered regularly interspaced short palindromic repeats (CRISPR)/CRISPR-associated (Cas) protein systems, genome editing and animal model production have been rapidly extended due to the ease and flexibility of these systems [2]. The CRISPR/Cas9 system, initially described as a protective response to phage infection, has been engineered as RNA-guided endonucleases that consist of a guide RNA (gRNA) and a Cas9 endonuclease. The gRNA binds to a specific DNA sequence with Cas9 [3]. After identifying the protospacer adjacent motif (PAM), which is marked by the 5ʹ-NGG-3ʹ sequence, and creating an RNA-DNA duplex, the Cas9 enzyme cuts the double-stranded DNA three base pairs before the PAM site [4]. The double-strand break (DSB) created by Cas9 can be repaired through either error-prone nonhomologous end joining (NHEJ) or high-fidelity homology-directed repair (HDR). The latter allows for accurate modification of the genome at the DSB site by utilizing a homologous repair template [5].

In 1989, sperm-mediated gene transfer (SMGT) emerged as a new approach to animal genetic manipulation. This technique relies on the capability of sperm to spontaneously absorb genes in laboratory conditions and deliver them to the oocyte during fertilization [6]. In the SMGT technique, the activity of the reverse transcriptase enzyme in sperm cells leads to the production of retrogenes, which can be transcribed and have the potential to generate new phenotypic traits in animals. Most SMGT protocols are expected to result in extrachromosomal arrangements, a low copy number, and mosaic distribution of foreign DNA and retrogenes among organs and within organisms [7]. These characteristics contribute to this technique’s low efficiency and reproducibility in creating transgenic animals [8–10]. Despite its limitations, the potential use of SMGT for targeted mutant embryo production via the CRISPR-Cas9 system is theoretically appealing. Unlike other techniques, the CRISPR system does not require integration into the genome to produce targeted knockout embryos. Additionally, the SMGT technique is simple and simultaneously enables genetic modification in multiple embryos. However, the incubation of spermatozoa with exogenous DNA alone is not efficient enough, leading researchers to develop various protocols to enhance the efficiency of SMGT. These enhancements include the use of lipofectamine [11], restriction enzyme-mediated insertion (REMI) [12], electroporation [13], linker-based technique [14], viral vectors [15], magnetic nanoparticles [16], ICSI-transgenesis [17, 18], and DMSO [19].

It is well-established through numerous studies that cholesterol has direct and indirect effects on the biophysical traits of biological membranes. Indirectly, it affects the orientation of the membrane lipids’ hydrocarbon chains, resulting in decreased membrane fluidity. Consequently, this decrease in fluidity hinders the ability of membrane proteins to undergo appropriate conformational changes, thereby inhibiting the function of many proteins and stabilizing the membrane. Moreover, cholesterol directly binds to membrane proteins, regulating their function, which can either stimulate or inhibit them [20]. Methyl β-cyclodextrin (MBCD) is in the family of β-cyclodextrins (βCDs), cyclic glucose heptamers. Structurally, CDs have an external hydrophilic surface and a central lipophilic cavity, which provides a small environment to contain and dissolve hydrophobic guest molecules. CDs, particularly MBCD, have the ability to interact with cell membranes and remove cholesterol and other lipids from them. This functionality can change the integrity and function of membrane domains, making it a commonly used tool for altering the structure and functionality of cell membranes in the field of cell biology [21]. In 1998, Choi and Toyoda were the first to use a protein-free c-TYH medium supplemented with 1 mg of PVA and 0.75 mM MBCD for the incubation of spermatozoa. They showed that similar to BSA, MBCD can remove non-esterified cholesterol from sperm cell membranes [22]. When MBCD is used, the removal of cholesterol occurs more rapidly and efficiently than with BSA, which can increase sperm fertility [23]. Additionally, the treatment of sperm cells with MBCD, unlike BSA, leads to the extraction of cholesterol from lipid-raft regions and induces changes in the activity of cholesterol-rich microdomains [24]. The amount of cholesterol released by MBCD depends on factors such as cell type, MBCD concentration, incubation time, and temperature [25]. Considering that many mechanisms of exogenous DNA uptake by sperm cells and the roles of cholesterol in this process are not yet fully understood and that effective delivery of the CRISPR-Cas9 system to create targeted mutant mice is a major challenge, we decided to optimize the uptake of this system into sperm cells while using sperm cells as a tool to transfer the CRISPR-Cas9 system to produce targeted mutant blastocysts and mice. In the current study, we assessed the effect of the c-TYH medium with different concentrations of MBCD on exogenous DNA uptake by murine spermatozoa, as well as the production efficiency of targeted mutant mouse blastocysts and offspring.

## Materials and methods

The present study received ethical and research committee approval from the veterinary faculty of Shiraz University (99GCB4M154630). Unless stated otherwise, all the chemicals used in this study were obtained from Sigma-Aldrich Chemical Co.

### Animals

Eight to 12-week-old B6D2F1 (C57/BL/6J×DBA/2J) and CD1 males and females were acquired from the Royan Institute (Tehran, Iran). They were fed a standard autoclaved mouse diet and were given unlimited sterilized tap water. A consistent light/dark cycle of 12 h of light and 12 h of darkness was maintained throughout the experiment.

### Media

The following media were used: (I) Human tubal fluid (HTF) (this medium was used for sperm incubation in control groups and fertilization in all groups). (II) Choi- Toyoda Yokoyama Hosi (c-TYH) supplemented with 0, 0.75, 1, and 2 mM concentration of MBCD (this medium was used for sperm incubation of treatment groups). (III) Modified potassium simplex optimization medium (mKSOM) (this medium was used for in vitro culture of fertilized eggs). (IV) Flushing holding medium (FHM) (this medium was used for oocyte collection). (V) Uterine transfer medium (UTM) (Cooper Surgical, Denmark) (this medium was used for embryo transfer).

### Preparation of exogenous gene constructs

This study used the plenti-CAG-gate-FLAG-IRES-GFP (Addgene plasmid #107398) (pCAG-GFP) and pCAG-eCaS9-GFP-U6-gRNA (Addgene plasmid #79145) (pgRNA-Cas9) vectors. The pCAG-GFP was used as a calibrator to exclude possible off-target effects of the CRISPR-Cas9 system on our experimental setup. The plasmids were cultivated in an Stbl4-competent bacterial strain through transformation and cultured in Luria-Bertani (LB) broth medium supplemented with kanamycin and ampicillin, respectively. Miniprep purification was performed using a plasmid extraction kit (Favorgen, Taiwan) based on the manufacturer’s guidelines. The single-guide RNA (sgRNA) was designed for the growth differentiation factor-8 (*Gdf8*) gene (NC_000067.7) using an online gRNA design tool. To determine unique target sites in the mouse genome, the guide strands were designed to target the first exon at position 184–203 base pairs. These strands were synthesized as sense and antisense oligodeoxynucleotides with a *BbsI*-specific sticky end at the 5’ terminus (sense strand sequence: 5’ CACCGGCCCAGTGGATCTAAATGA 3’, and antisense strand sequence: 5’ AAACTCATTTAGATCCACTGGGCC 3’). Subsequently, the annealed complementary oligos were ligated with T4 DNA ligase into digested pgRNA-Cas9 vectors that were linearized with the *BbsI* enzyme. The resulting structure was then propagated in an stbl4-competent bacterial strain. To ensure correct ligation, the insert sequence was validated through colony PCR using the following primers: forward primer 5’ GATACAAGGCTGTTAGAGAG 3’ (U6-F) and reverse primer 5’ AAACTCATTTAGATCCACTGGGCC 3’ (antisense gRNA) and confirmed by Sanger sequencing. The confirmed colonies were then cultivated, and plasmid DNA was extracted. The concentration and quality of the purified plasmid were evaluated using a Nanodrop spectrophotometer (Thermo Scientific, USA) as well as 0.8% agarose gel electrophoresis. Finally, this plasmid was utilized for further investigations.

### Study design

In the present study, we classified our experimental groups into three major categories based on the content of the sperm incubation medium: control, sham, and treatment groups. The control group was further subdivided into a negative control group and a positive control group. We used the DMSO-SMGT protocol as a positive control, an improved and commonly used method for SMGT, and compared the results of the treatment groups with it. Based on the type of plasmid used, the positive control group was divided into the DMSO/CAG-GFP and DMSO/gRNA-Cas9 groups. Based on MBCD concentration, the sham group was divided into four groups (0, 0.75, 1, and 2 mM MBCD). Based on the type of plasmid used, the treatment group was divided into CAG-GFP and gRNA-Cas9 groups. Based on MBCD concentration, the last two groups were divided into three groups (0.75, 1, and 2 mM MBCD).

**Fig. 1:**
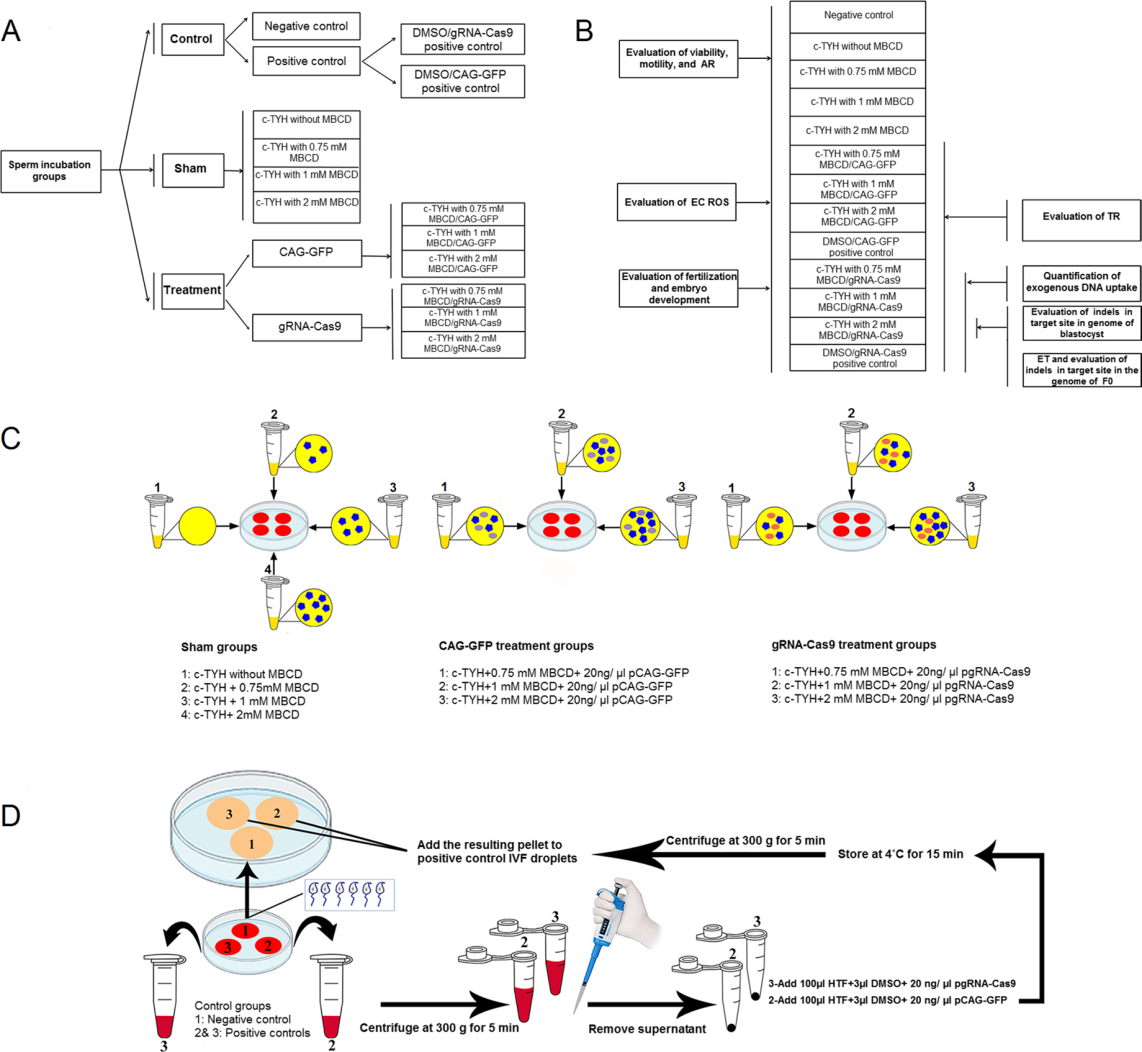
Study design. **A** Classification of experimental groups. In the negative and positive control groups, sperm were incubated in HTF + 0.4% BSA for 30 min. After 30 min, sperm in DMSO/CAG-GFP and DMSO/gRNA-Cas9 groups were treated with 3% DMSO and 20 ng/µl plasmid for 15 min at 4 °C. In the sham groups, sperm were incubated in a c-TYH medium with different concentrations of MBCD (0, 0.75, 1, and 2 mM) for 30 min. In the treatment groups, sperm were incubated in a c-TYH medium with different concentrations of MBCD (0.75, 1, and 2 mM) in the presence of 20 ng/µl plasmids for 30 min. **B** Overview of assessment performed on experimental groups. **C** Details of the sperm incubation medium for sham and treatment groups. **D** Schematic view of DMSO treatment of sperm. EC: extracellular; TR: transfection rate

In the negative and both positive control groups, sperm were incubated in an HTF medium supplemented with 0.4% BSA. However, after 30 min of incubation at 37 °C in 7.5% CO2, in both DMSO/CAG-GFP and DMSO/gRNA-Cas9 positive control groups, spermatozoa were added to HTF medium supplemented with 0.4% BSA, 3% DMSO, and 20 ng/µl pCAG-GFP or pgRNA-Cas9, respectively, and incubated at 4 °C for 15 min. In the sham groups, sperm were incubated in c-TYH medium with 0, 0.75, 1, and 2 mM MBCD at 37 °C in 7.5% CO2 for 30 min. In the treatment groups, sperm were incubated in a c-TYH medium with different concentrations of MBCD (0.75, 1, and 2 mM) in the presence of plasmids, either 20 ng/µl pCAG-GFP or pgRNA-Cas9, at 37 °C in 7.5% CO2 for 30 min. The study design of this investigation is presented in Fig. 1.

### Gamete preparation and co-incubation

#### Oocyte collection

Superovulation was induced in female B6D2F1 mice by administering a 10 IU intraperitoneal injection of pregnant mare serum gonadotropin (PMSG) (Gonaser: Laboratories Hipra, Spain). Fifty hours later, 10 IU of human chorionic gonadotropin (hCG) (PDpreg: Pooyeshdarou Co., Iran) was injected intraperitoneally. Fourteen hours after the hCG injection, the females were sacrificed by cervical dislocation. The oviducts were then removed and placed in a dish containing FHM medium. Subsequently, cumulus-oocyte complexes (COCs) were collected from the ampullary region of the oviducts. The collected COCs were washed and placed in HTF-containing IVF dishes covered with mineral oil.

#### Sperm preparation and sperm-oocyte co-incubation

Male B6D2F1 mice were sacrificed by cervical dislocation to obtain the caudal epididymis and deference ducts. Two media were used for collecting spermatozoa: HTF medium supplemented with 0.4% BSA and c-TYH medium without MBCD. Both media were incubated at 37 °C in 7.5% CO2 for 20 min prior to use. The c-TYH medium without MBCD was used for sperm collection in the sham and treatment groups, while the HTF medium was used for the control groups. Spermatozoa were released by mechanically dissecting the caudal epididymis and squeezing the deference ducts, after which the collected sperm were added to the related sperm incubation medium. Details of each group and sperm incubation medium are shown in Fig. 1. In all groups except the DMSO/CAG-GFP and DMSO/gRNA-Cas9 positive control groups, the incubated spermatozoa were added to the related HTF-containing IVF droplets after 30 min incubation at 37 °C in 7.5% CO2 in the sperm incubation medium. In the DMSO/CAG-GFP and DMSO/gRNA-Cas9 positive control groups, the incubated spermatozoa were collected into two microtubes and centrifuged. The resulting pellets from each microtube were added to another microtube containing HTF medium supplemented with 0.4% BSA, 3% DMSO, and either 20 ng/µl of pCAG-GFP or 20 ng/µl of pgRNA-Cas9, respectively. These microtubes were then incubated at 4 °C for 15 min (Fig. 1D). Finally, the samples were centrifuged, and each resulting pellet was added to the related HTF-containing IVF droplet.

### Experiment 1

#### Assessment of sperm motility, viability, and acrosomal reaction

To assess sperm motility, 10 µl sperm samples were loaded onto a slide after 30 min of incubation. A coverslip was then placed on the slide, and an assessment was performed at a ×400 magnification under bright-field microscopy. Each spermatozoon was classified into one of three motility categories: progressive, non-progressive, or immotile. Eosin Y staining was utilized to evaluate the viability of sperm. Briefly, after 30 min of incubation, 10 µl of each sperm sample was transferred onto a microscope slide using a pipette. Next, the samples were mixed with 10 µl of a staining solution (0.67% eosin Y dissolved in 0.9% normal saline). The mixture was then incubated for 30 s at room temperature (20 °C). Subsequently, a coverslip was placed on the slide, and counting was performed at a ×400 magnification under bright-field microscopy. Sperm that appeared colorless were categorized as alive, while those displaying any shade of pink or red were categorized as dead. Afterward, Coomassie Brilliant Blue (G-250) was used to assess the acrosomal reaction. After 30 min of incubation, sperm samples were fixed with a 4% paraformaldehyde solution (pH 7.4) for 10 min at 24 °C. Sperm were then centrifuged and washed twice with PBS. The resulting sperm pellet was resuspended in PBS, and 30 µl of the sperm suspension was spread on glass microscope slides using another glass slide and then left to dry in the air. Slides were subsequently dipped in a recently prepared coomassie stain (0.22% Coomassie Blue G-250, 50% methanol, 10% glacial acetic acid, 40% water) for two minutes. Following staining, the slides were thoroughly washed with distilled water to eliminate excess stain. Finally, the slides were left to dry and the stained spermatozoa were observed using bright-field microscopy at a ×1000 magnification with oil immersion.

### Experiment 2

#### Luminol-dependent chemiluminescence assay (LDCA) in sperm incubation media for assessment of extracellular ROS

Luminol (5-amine-2, 3-dihydro-1, 4-phtalazinedione) interacts with various extracellular and intracellular reactive oxygen species (ROS) at neutral pH. A luminometer converts the light signals generated by the interaction of luminol and free radicals into electrical signals in the form of photons. Relative Light Units (RLU) are used to quantify the amount of ROS. To prepare a 100 mM stock solution of luminol, 0.0089 g of luminol was carefully weighed and then dissolved in 500 µl of DMSO in a microtube covered with aluminum foil to protect the luminol from being exposed to light, as it is sensitive to light. Subsequently, to prepare a 5 mM working luminol solution, 20 µl of the luminol stock solution were combined with 380 µl of DMSO in a microtube covered with foil.

LDCL assays of sperm incubation media were performed using a luminometer. Briefly, all the sperm incubation droplets were first collected separately and centrifuged at 300 g for five minutes. The supernatant was then collected, and 2.5 µl of a working luminol solution was added to 100 µl of each sample. Additionally, blank, positive, and negative control samples were prepared as follows: for the blank sample, 100 µl of PBS; for the positive control, 100 µl of PBS + 12.5 µl of H2O2 30% (V/V) + 2.5 µl of working luminol solution; and for the negative control, 100 µl of PBS + 2.5 µl of working luminol solution. Immediately after preparation, all samples were moved into a 96-well, untreated black polystyrene plate. Three replicates were considered for each sample. The LDCL was measured using an Orion II LB 965 Microplate Luminometer (EG & G Berthold, Germany) at 37 °C. The LDCL assay was performed 15 times with a read time of one second and an interval of one minute (total time: 900 s). The RLU mean was calculated as the RLU/s mean × 900 s for each sample. Then, the ROS level of each sample was calculated using the following formula: ROS level of the sample = RLU mean of the sample- RLU mean of the negative control.

### Experiment 3

#### Quantification of exogenous DNA uptake by sperm using absolute real-time PCR technique

For this assessment, sperm were incubated in the c-TYH medium with different concentrations of MBCD (0, 0.75, 1, and 2 mM) in the presence of 20 ng/µl pgRNA-Cas9. Also, sperm from both the DMSO/gRNA-Cas9 positive and negative control groups were used. To extract both genomic DNA and exogenous DNA from the treated sperm, a tissue genomic DNA extraction mini kit (Favorgen, Taiwan) with a specific sperm DNA extraction protocol was used. Following sperm incubation, spermatozoa from the mentioned groups were collected in microtubes, centrifuged at 300 g, and washed twice in base medium (HTF for the negative and positive control groups and c-TYH for the others) to eliminate any exogenous DNA that was not internalized or attached to the surface of the sperm membrane. To remove attached but uninternalized exogenous DNA from spermatozoa, the samples were incubated with 10 IU of DNase I in 100 µl of base medium for 30 minutes at 37°C, followed by two washes with the base medium. For the deactivation of DNase I, 0.002 g of EDTA was added to 190 µl of the base medium. This solution was then combined with 10 µl of the sperm pellet. Subsequently, the mixture was centrifuged, and the sperm pellet was used for sperm DNA extraction based on the kit protocol. The extracted DNA was evaluated and quantified using 1% agarose gel electrophoresis and a NanoDrop spectrophotometer (Thermo Scientific, USA). Finally, the DNA samples were preserved at -20°C until further use. To confirm the internalization of the plasmid, the conventional PCR technique was used (primer F: 5’ GATACAAGGCTGTTAGAGAG 3’ (U6-F) and primer R: 5’ AAACTCATTTAGATCCACTGGGCC 3’ (antisense gRNA)). The conventional PCR was performed in a Mastercycler Gradient thermal cycler, and the amplification conditions were one denaturation step at 95 °C for 5 min, 40 cycles at 95 °C for 30 s, 60 °C for 30 s, and 72 °C for 45 s, followed by a final extension for 10 min at 72 °C. Subsequently, absolute real-time PCR was used to assess the copy numbers of exogenous DNA internalized per sperm cell. This approach is the best technique for measuring the uptake of exogenous DNA by spermatozoa [9, 26–28].

The standard curves for the genomic DNA of spermatozoa and the used plasmid were generated by preparing 10^4^, 10^3^, 10^2^, and 10^1^ copies of genomic DNA and 10^6^, 10^5^, 10^4^, 10^3^, 10^2^, and 10^1^ copies of the plasmid. Copy numbers of the genomic DNA extracted from the sperm sample of the negative control and the copy numbers of the plasmid sample were calculated with the copy number calculator tool (https://www.technologynetworks.com/tn/tools/copynumbercalculator). Copy number calculation requires two values: the length of the template in bp (the length of mouse sperm genomic DNA and plasmid: 2.9 × 10^9^ and 10,245 bp, respectively) and DNA concentration (ng/µl). DNA concentration was measured using a NanoDrop spectrophotometer (Thermo Scientific, USA), and DNA purity was confirmed by assessing the 260/280 nm ratio.

The genomic DNA standard curve was created to quantify the number of sperm. This curve was generated using specific primers designed to target the murine β-actin gene (*Actb*), which exists in a single copy within a haploid genome. Primer-blast analysis was used to eliminate the amplification of pseudogenes and closely related sequences (http://www.ncbi.nlm.nih.gov/tools/primer-blast/primerinfo.html). The forward primer sequence for the *Actb* gene is 5’ CCACCATGTACCCAGGCATT 3’, while the reverse primer sequence is 5’ CCCACCCTCACCAAGCTAAG 3’. The plasmid DNA standard curve was created to quantify the plasmid copy numbers using primers designed to target the specific sequence of the plasmid being used. The forward primer used for detecting the plasmid (U6-F) had the sequence 5’ GATACAAGGCTGTTAGAGAG 3’, while the reverse primer (antisense gRNA) had the sequence 5’ AAACTCATTTAGATCCACTGGGCC 3’. The quantitative analysis was performed using the Step One Plus 96-Well Real-Time PCR system (Applied Biosystems, USA) and the qPCR Master Mix containing SYBR Green® (2x) (Yekta Tajhiz Azma, Iran). In each reaction, the mixture comprised 6.5 µl of master mix, 0.25 µl of ROX dye (50x), 0.25 µl of 10 µM of each primer, 5 µl of Distilled water, and 1 µl of DNA template (13 ng), resulting in a total volume of 13 µl. Reactions were prepared in triplicate for each run. The real-time PCR procedure involved a single initial step at a temperature of 95 °C for two minutes. This was followed by 40 amplification cycles, consisting of five seconds at 95 °C and 30 s at 60 °C. Subsequently, a melting curve stage was conducted, comprising 15 s at 95 °C, one minute at 60 °C, and another 15 s at 95 °C. The data acquisition, standard curve fittings, and analysis were carried out using Step One Software v2.3.

### Experiment 4

#### Assessment of fertilization, embryo development, and transfection rate in SMGT-derived blastocysts

After six hours of adding sperm cells to IVF droplets, the presumptive zygotes were washed and transferred to mKSOMaa-containing IVC droplets. After 96 h, early embryonic development was assessed using a stereo microscope, and the percentage of fertilization (presence of two pronuclei, 2PN), two-cell, four-cell, compacted morula, and blastocyst stage embryos were recorded. The GFP-positive blastocysts of CAG-GFP, gRNA-Cas9, and positive control groups were detected under a fluorescence microscope, and the rate of GFP-positive blastocysts was determined.

### Experiment 5

#### Evaluation of CRISPR-Cas9 system efficiency and tracking of targeted small insertion or deletion (indel) mutations in MBCD-SMGE derived blastocysts by sanger sequencing

In order to obtain the CRISPR-Cas9 efficiency rate, four GFP-positive blastocysts from the 2 mM MBCD gRNA-Cas9 group were selected. For genomic DNA extraction of blastocysts, each blastocyst was placed into a microtube containing 9 µl of lysis buffer composed of 10 mM Tris-HCL (PH = 8), 1.5 mM MgCl2, 50 mM KCl, 0.5% (V/V) Tween-20, 0.5% (V/V) IGEPAL, and 100 µg/cc proteinase K and incubated for 60 minutes at 65°C, followed by 10 minutes at 95°C. Finally, the content of each microtube was used as the PCR template. The target site in the desired gene (*Gdf8*) was isolated using the PCR technique with the following primers: *Gdf8* forward primer 5’ AGAAGAACGGCATCAAGG 3’ and *Gdf8* reverse primer 5’ GCTCAGGTAGTGGTTGTC 3’. The conventional PCR was carried out using a Mastercycler Gradient thermal cycler (Eppendorf, Germany) with the following conditions: one denaturation step at 95 °C for 5 min, followed by 40 cycles of denaturation at 95 °C for 30 s, annealing at 58 °C for 45 s, and extension at 72 °C for 45 s. A final extension step was performed at 72 °C for 10 min. To check the specificity and size of PCR products, 2% agarose gel electrophoresis was performed. Subsequently, the Sanger sequencing process was performed on the PCR products, and the resulting data were analyzed to detect any indels at the target site. This analysis was performed using the trace decomposition method through the inference of CRISPR edits (ICE) software (https://ice.synthego.com).

### Experiment 6

#### Embryo Transfer (ET) and targeted mutant mouse production

In this study, vaginal plug (VP)-positive pregnant mice at 0.5 days post-coitus (dpc) and blastocysts derived from the 2 mM MBCD gRNA-Cas9 group were used for ET. In parallel with IVF, on the same day of IVF, the CD1 females, kept in groups of eight females in cages away from males for two weeks, were taken into the male cage substrate. On the third day after IVF, they were transferred to the cage of male CD1 mice (male-to-female ratio: 1:1). On the fourth day after IVF, the presence of VP was checked in the female mice, and VP-positive mice were separated for ET. ET was performed under general anesthesia in a ventral recumbency position with an incision site 0.5 cm above the hip joint at the midline. The peritoneum was exposed, followed by incisions on the left and right sides of the peritoneum, approximately 0.5 cm away from the vertebral column. The ovary and oviduct were exposed, and a small incision was made on the ovarian bursa. Approximately 10 blastocysts obtained from IVF in BDF1 mice were inserted into each oviduct through the infundibulum using a mouth pipette and minimal culture medium (UTM™ medium was used for ET). Then, the peritoneum and skin were sutured with 6 − 0 non-absorbable sutures using a simple interrupted method. After recovery, the mouse was transferred back to its cage and observed for pup birth after 19 days. Dark-coated pups were identified among the white-coated ones after one week. After three weeks, one of the dark-coated mice was separated from the surrogate mother and euthanized for genome extraction, plasmid presence investigation, and target sequence evaluation. Genome extraction was performed using the tissue genomic DNA extraction kit (Favorgen, Taiwan) according to the kit’s instructions, and the quality of the extracted products was analyzed using a 1% agarose gel. To examine the presence of the gRNA-Cas9 plasmid in the genome of the dark-coated mouse, PCR was performed on genomic DNA extracted from kidney, liver, brain, and muscle using a forward primer (U6-F): 5’ GATACAAGGCTGTTAGAGAG 3’ and a reverse primer (antisense gRNA): 5’ AAACTCATTTAGATCCACTGGGCC 3’. Subsequently, to assess the evidence of the indels at the target site in the extracted genomic DNA from the mentioned tissues, the target site in the desired gene (*Gdf8*) was isolated by PCR technique and sequenced, as explained in experiment 5. The sequencing results were analyzed by ICE software.

### Statistical analysis

In the figure legends and table footnotes, n represents the number of independent biological replicates. The raw data were analyzed using the Shapiro-Wilk test to evaluate the distribution of the data and the Levene test to assess variance homogeneity. SPSS software (version 23) was used to apply these procedures in all analyses. The mean ± standard error of the mean (SEM) was utilized to present the results. In cases where the assumptions were true, one-way analysis of variance (one-way ANOVA) and Tukey’s *Post Hoc* test were used (for variables such as progressive motility, viability, and acrosomal reaction, GFP-positive blastocysts rate), and statistical significance was established when *P* < 0.05. In cases where the data were normal but the homogeneity of variances was not established, the Games-Howell test was employed as a *Post Hoc* test (for variables such as fertilization rate, copy number of plasmid/ sperm cell, and ROS level). The rates of the four-cell embryo, compacted embryo, and blastocyst were analyzed using the Kruskal-Wallis test.

## Results

### Experiment 1

#### Motility, viability, and acrosome integrity were altered in spermatozoa incubated with MBCD and DMSO

For evaluating the effect of experimental treatments on spermatozoa, motility, viability, and acrosome status were assessed. The results of these analyses are shown in Table 1. Progressive motility and viability were decreased in the c-TYH medium supplemented with 2 mM MBCD (51% and 53.33%, respectively; *P* < 0.05). Additionally, incubation of sperm with DMSO at 4 °C severely reduced motility, viability, and acrosomal integrity (*p* < 0.05). No significant differences were observed in motility, viability, or acrosome reaction between the sham and treatment groups. Furthermore, the introduction of different plasmid constructs at a concentration of 20 ng/µl into the sperm incubation media did not affect the functional parameters of the sperm. Generally, incubation of sperm with c-TYH medium for 30 min decreased viability compared to the negative control group. Moreover, the presence of MBCD significantly increased the acrosomal reaction.

**Table 1:** Effect of the experimental treatments on functional parameters of sperm cells and extracellular ROS levels

Group	Progressive Motility (%)	Viability (%)	Acrosomal Reaction (%)	Extracellular ROS level (RLU)
HTF + 0.4%BSA	78.33 ± 0.88^a^	89 ± 0.58^a^	5 ± 0.45^a^	415 ± 35^a^
c-TYH + 0mM MBCD	77.67 ± 1.45^a^	79.67 ± 0.88^b^	4.5 ± 0.5^a^	181 ± 19^a^
c-TYH + 0.75mM MBCD	73.67 ± 1.86^a^	76.33 ± 1.45^b^	12.33 ± 1.2^b^	945 ± 45^b^
c-TYH + .75mM MBCD + 20ng/ul pCAG-GFP	76.33 ± 2.03^a^	78 ± 1.16^b^	12.67 ± 0.88^b^	930 ± 30^b^
c-TYH + .75mM MBCD + 20ng/ul pgRNA-Cas9	77 ± 1.53^a^	78 ± 0.58^b^	12 ± 1.23^b^	950 ± 50^b^
c-TYH + 1mM MBCD	71.33 ± 1.33^a^	74 ± 0.58^b^	15 ± 0.71^b^	1189 ± 10.5^b^
c-TYH + 1mM MBCD + 20ng/ul pCAG-GFP	73 ± 1^a^	75 ± 0.58^b^	16.33 ± 1.2^b^	1160 ± 15^b^
c-TYH + 1mM MBCD + 20ng/ul pgRNA-Cas9	73.33 ± 1.2^a^	75.67 ± 0.88^b^	15.67 ± 1.33^b^	1201.6 ± 20^b^
c-TYH + 2mM MBCD	51 ± 1.53^b^	53.33 ± 1.76^c^	23 ± 1.08^c^	3319.5 ± 19.5^c^
c-TYH + 2mM MBCD + 20ng/ul pCAG-GFP	51 ± 0.58^b^	53 ± 0.33^c^	21.67 ± 0.33^c^	3330.2 ± 17^c^
c-TYH + 2mM MBCD + 20ng/ul pgRNA-Cas9	50.33 ± 0.33^b^	53 ± 0.58^c^	21.33 ± 0.88^c^	3350 ± 10^c^
HTF + 0.4%BSA + 3%DMSO + 20ng/ul pCAG-GFP	11.67 ± 0.88^c^	44 ± 0.33^d^	20.33 ± 0.33^c^	3400 ± 25^c^
HTF + 0.4% BSA + 3% DMSO + 20ng/ul pgRNA-Cas9	12.67 ± 1.45^c^	45 ± 2.19^d^	22 ± 1^c^	3451 ± 49^c^

Relative Light Units (RLU) are used to quantify the amount of ROS.

Data are shown as mean ± SEM of three replicates (*n* = 3)

^a–d^ significant differences (*p* < 0.05) exist between values in the same column when accompanied by different superscripts

The functional parameters of sperm were analyzed through a one-way ANOVA statistical test to compare different groups. Pairwise comparisons were conducted using Tukey’s *Post Hoc* test. Additionally, the levels of extracellular ROS were evaluated through a one-way ANOVA, followed by the Games-Howell *Post Hoc* test

### Experiment 2

#### Extracellular ROS were greatly increased in c-TYH medium containing 2 mM MBCD

We evaluated the effects of incubating sperm at different concentrations of MBCD in the presence or absence of exogenous DNA on extracellular ROS levels. These results were then compared to the extracellular ROS levels observed in the positive and negative control groups. The results are shown in Table 1. Incubation of sperm with MBCD and DMSO resulted in a significant increase in the extracellular ROS level (*P* < 0.05). The introduction of exogenous DNA into sperm at a concentration of 20 ng/µl did not affect the level of extracellular ROS. No significant difference in ROS levels was observed between the c-TYH medium without MBCD and the negative control group (*p* > 0.05).

### Experiment 3

#### The rate of exogenous DNA internalization into sperm in the c-TYH medium was MBCD dose-dependent

**Fig. 2:**
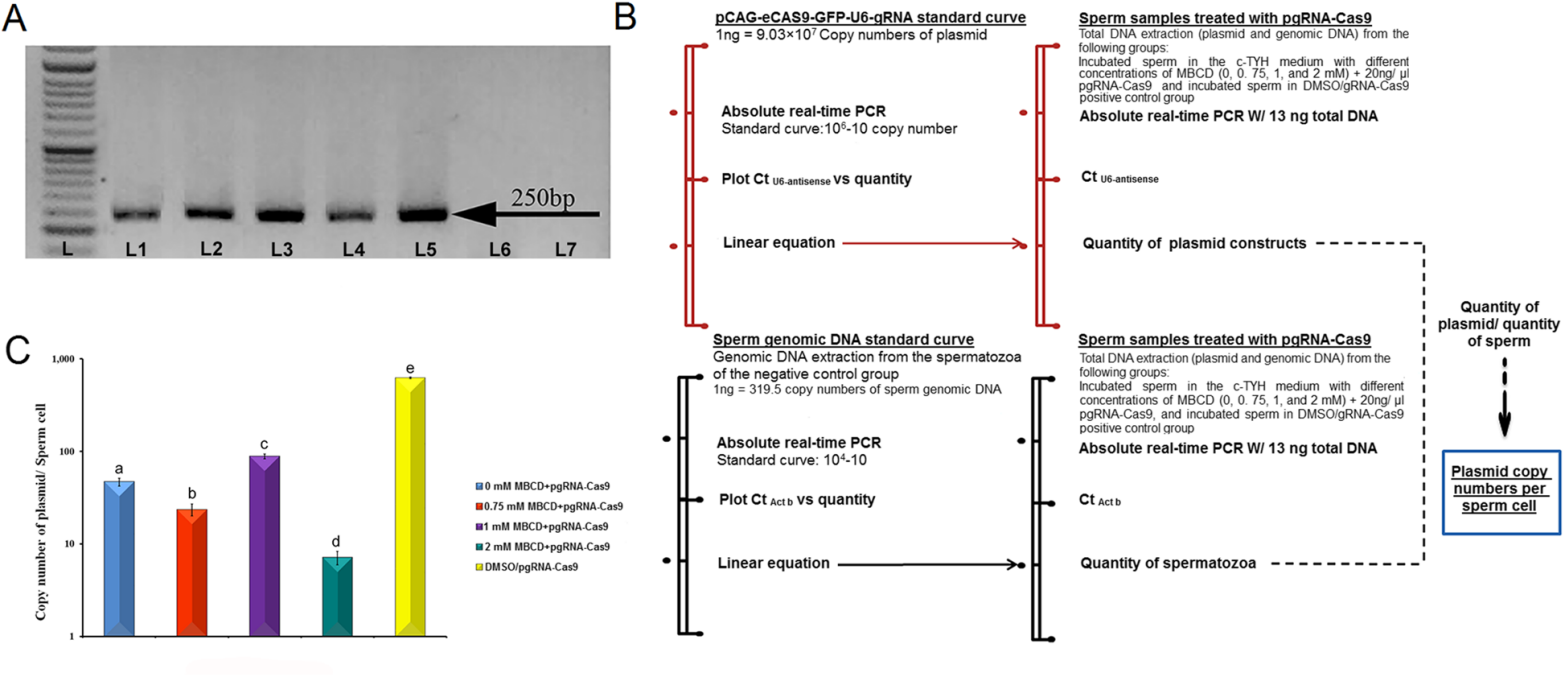
Identification and quantification of exogenous DNA uptake by sperm using conventional PCR and absolute real-time PCR. Total DNA was extracted from sperm from experimental groups treated with pgRNA-Cas9 after removing unbound or non-internalized plasmids. **A** Conventional PCR and gel electrophoresis were performed to confirm plasmid internalization into sperm. Gel electrophoresis detected fragments of 250 bp between the U6 promoter and the guide region. L: 50 bp DNA ladder; L1-L4: 0, 0.75, 1, and 2 mM MBCD + pgRNA-Cas9 groups, respectively; L5: DMSO/gRNA-Cas9 group; L6: negative control group (HTF + 0.4%BSA); and L7: NTC. **B** The diagram illustrates the method employed to determine the copy number of internalized plasmid per sperm. **C** Quantification of plasmid copy numbers internalized per sperm cell using absolute real-time PCR. The data are shown as mean ± SEM of two independent experiments performed in triplicate (*n* = 2). Differences between means with a *p* < 0.05 are indicated by different small letters. A one-way ANOVA statistical test was used to compare the groups, and a Games-Howell *Post Hoc* test was performed for pairwise comparisons. NTC: No template control

We used absolute real-time PCR to determine the copy numbers of plasmid constructs internalized per sperm cell in each experimental group. Before performing absolute real-time PCR, the presence of plasmid in the treated samples was confirmed through conventional PCR (Fig. 2). The incubation of sperm with different concentrations of MBCD resulted in a concentration-dependent alteration in the copy numbers of plasmid uptake per sperm cell. In the absence of MBCD, 46.85 ± 2.6 copies per sperm cell were internalized. However, in the presence of 0.75 mM MBCD, the amount of plasmid uptake by sperm was significantly decreased (*P* < 0.05), and 23.53 ± 1.71 copies per sperm cell were internalized. At the 1 mM MBCD concentration, the sperm’s rate of plasmid uptake was significantly increased (88.36 ± 3.79; *P* < 0.05), but at the 2 mM MBCD concentration, the amount of plasmid internalization was decreased (7.14 ± 0.69; *P* < 0.05). It is worth noting that incubating sperm with DMSO resulted in the internalization of a high copy number of plasmid constructs per sperm cell (624.18 ± 8.19; *P* < 0.05).

### Experiment 4

#### In vitro embryonic development was not affected by incubating sperm cells with MBCD and DMSO, while the production yield of GFP-positive blastocysts demonstrated an increase in the c-TYH medium

**Fig. 3:**
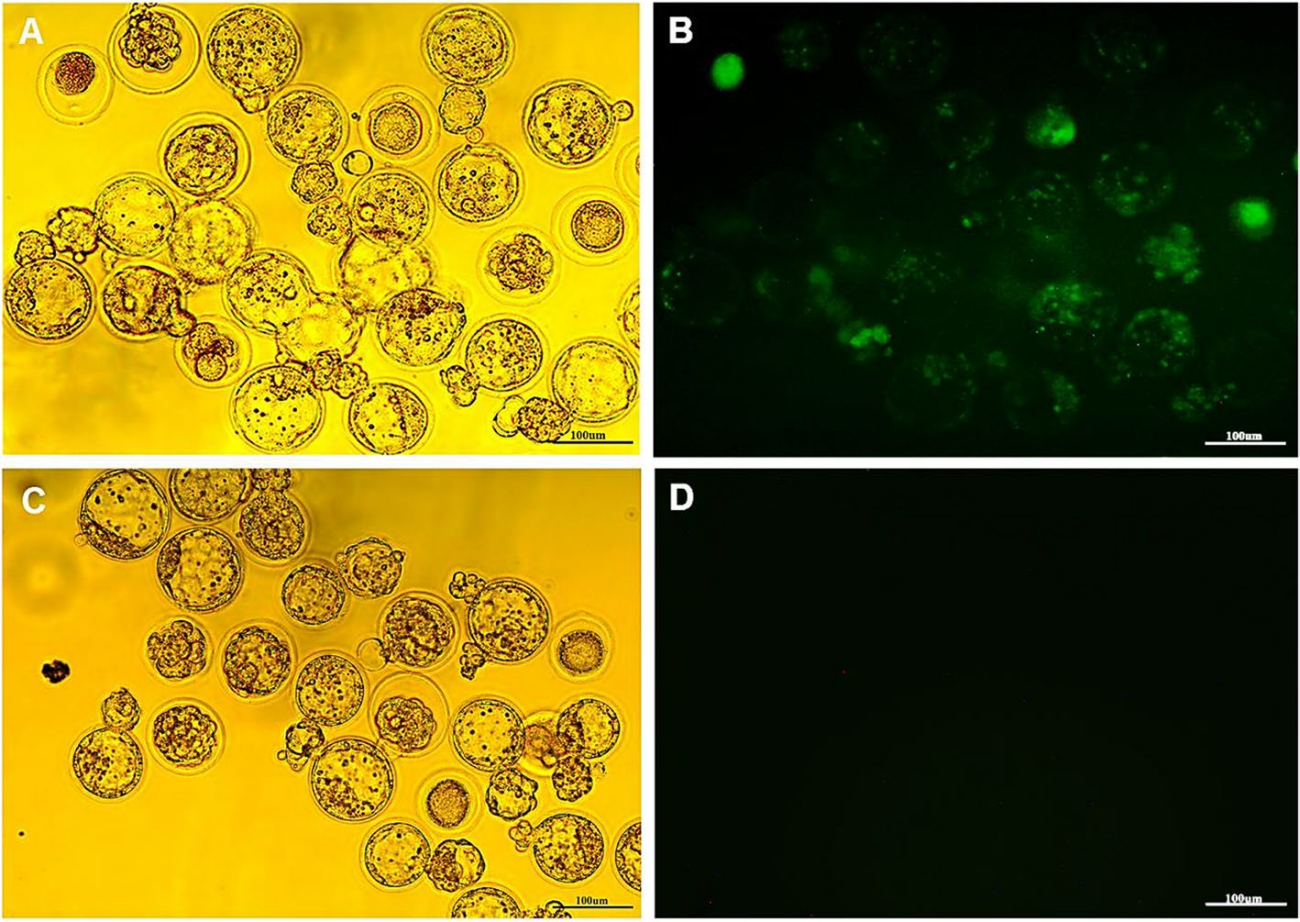
Murine GFP-positive blastocyst production using the MBCD-SMGE and IVF-IVC methods. **A** Bright field and (**B**) fluorescent field images of day 4 mouse blastocysts obtained from the MBCD-SMGE and conventional IVF-IVC methods. In the MBCD-SMGE method, sperm were incubated in the c-TYH medium supplemented with 2 mM MBCD and 20 ng/µl pgRNA-Cas9. **C** Bright field and (**D**) fluorescent field images of day 4 mouse negative control blastocysts. Scale bar size: 100 μm

The results of the evaluation of early embryonic development are shown in Table 2. Fertilization did not occur when sperm cells were incubated in the c-TYH medium without MBCD. No significant differences were observed in early embryonic development between the experimental groups (*p* > 0.05). On the other hand, the production rate of GFP-positive blastocysts was significantly higher when sperm cells were incubated in c-TYH medium with 0.75, 1, and 2 mM MBCD compared to the DMSO protocol (*p* < 0.05). Bright and fluorescent field images of day 4 blastocysts obtained from the MBCD-SMGE are shown in Fig. 3.

**Table 2:** Effect of the experimental treatments on in vitro embryonic development

Treatments	Oocyte no.	Fertilization rate%	Four-cell rate%	Compacted embryo rate%	Blastocyst rate%	GFP-positive blastocyst rate ^1^%
c-TYH + 0mM MBCD	125	0^a^	ND	ND	ND	ND
c-TYH + 0.75mM MBCD	117	91.50 ± 0.72^b^	94.67 ± 1.97^a^	93.81 ± 0.8^a^	91.78 ± 2.71^a^	ND
c-TYH + 1mM MBCD	127	93.02 ± 1.72^b^	94.86 ± 6.63^a^	88.75 ± 4.36^a^	86.68 ± 3.40^a^	ND
c-TYH + 2mM MBCD	121	95.21 ± 4^b^	98 ± 4^a^	95.68 ± 1.76^a^	92.64 ± 3.35^a^	ND
c-TYH + .75mM MBCD + 20ng/ µl pCAG-GFP	134	81.91 ± 7.78^b^	99.11 ± 1.79^a^	97.54 ± 3.04^a^	88.46 ± 4^a^	74.72 ± 6.98^a^
c-TYH + 1mM MBCD + 20ng/ µl pCAG-GFP	154	83.55 ± 11.81^b^	97.34 ± 3.51^a^	97.34 ± 1.31^a^	91.54 ± 1.75^a^	78.92 ± 6.10^a^
c-TYH + 2mM MBCD + 20ng/ µl pCAG-GFP	153	91.72 ± 1.5^b^	96.36 ± 2.62^a^	96.36 ± 2.62^a^	94.64 ± 2.11^a^	85.46 ± 3.54^a^
c-TYH + .75mM MBCD + 20ng/ µl pgRNA-Cas9	173	94.12 ± 1.57^b^	95.33 ± 7.80^a^	95.33 ± 3.19^a^	95.33 ± 3.19^a^	82.68 ± 10.19^a^
c-TYH + 1mM MBCD + 20ng/ µl pgRNA-Cas9	195	92.03 ± 3.39^b^	95.28 ± 6.53^a^	93.15 ± 3.7^a^	90.70 ± 4.67^a^	86.86 ± 6.79^a^
c-TYH + 2mM MBCD + 20ng/ µl pgRNA-Cas9	231	92.69 ± 1.19^b^	97.92 ± 3.79^a^	95.65 ± 1.61^a^	90.79 ± 4.13^a^	91.64 ± 3.6^a^
HTF + 0.4%BSA + 3%DMSO + 20ng/ µl pCAG-GFP	121	86.75 ± 6.17^b^	97.73 ± 4.55^a^	97.5 ± 2.5^a^	97.5 ± 2.5^a^	48.11 ± 4.66^b^
HTF + 0.4% BSA + 3% DMSO + 20ng/ µl pgRNA-Cas9	179	86.55 ± 3.24^b^	97.37 ± 6.45^a^	94.63 ± 3.63^a^	90.70 ± 4.38^a^	42.56 ± 1.74^b^
HTF + 0.4%BSA	140	89.07 ± 3.54^b^	98.90 ± 1.51^a^	97.44 ± 1.17^a^	97.5 ± 1.17^a^	ND

^1^ GFP-positive blastocyst rate (described as the total number of GFP-positive blastocyst/total number of blastocyst)

ND: not determined as the fertilization did not occur or no plasmid constructs were present in the sperm incubation medium

Data are shown as mean ± SEM of five replicates (*n* = 5). Fertilization rate was assessed using a one-way ANOVA followed by the Games-Howell *Post Hoc* test. The rates of a four-cell embryo, compacted embryo, and blastocyst were analyzed using the Kruskal-Wallis test. The rate of GFP-positive blastocyst was assessed using a one-way ANOVA followed by Tukey’s *Post Hoc* test

^a,b^ significant differences (*p* < 0.05) exist between values in the same column when accompanied by different superscripts

### Experiment 5

#### Targeted indel was validated in blastocysts derived from sperm incubated with 2 mM MBCD and 20 ng/µl gRNA-Cas9 plasmid constructs

**Fig. 4:**
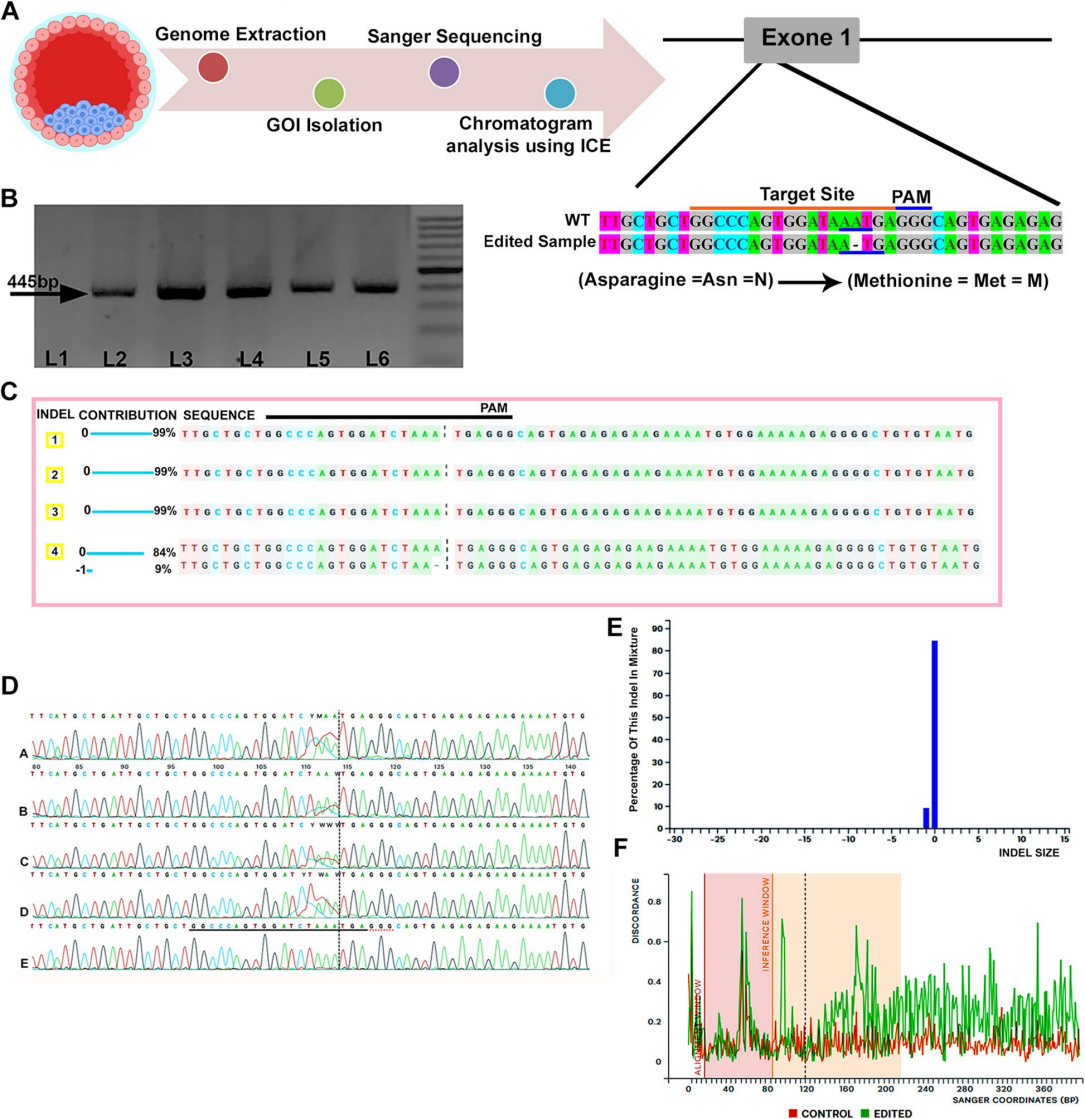
Validation of indel in MBCD-SMGE derived blastocyst by sequence trace decomposition method using inference of CRISPR edits (ICE) software. **A** Overview of the tracking process of targeted indel mutations in MBCD-SMGE derived blastocysts. **B** Gel-electrogram image shows isolated GOI from GFP-positive blastocyst obtained from sperm incubated in c-TYH with 2 mM MBCD and 20 ng/µl pgRNA-Cas9 (L2–L5) and control blastocyst (L6). L1: No-template control, and L: 100 bp DNA ladder. C-F: Output of computational analysis by ICE. **C** The relative contribution of the wild-type and edited sequence in four GFP-positive blastocyst samples (1-3: non-edited samples, 4: edited sample). **D** Sanger sequencing view displays the edited (a), non-edited (b, c, and d), and wild-type (control) sequences in the vicinity of the guide region. The guide sequence is represented by the black region that is underlined horizontally. **E** The indel plot of the edited blastocyst reveals the estimated distribution of indels across the entire genome of the blastocyst. **F** The discordance plot of the edited blastocyst provides details about the degree of similarity per base between the wild-type (control) and the edited sample within the specific region surrounding the cut site (inference window)

Sanger sequencing was performed to test whether the pgRNA-Cas9 can cleave the target site in blastocysts. For Sanger sequencing, the genomes of four blastocysts were first extracted, and then the desired fragment was isolated by PCR (Fig. 4). Lastly, PCR products were directly sequenced. Computational analysis of the resulting chromatograms using ICE software showed targeted cleavage in one of four samples (CRISPR/Cas9 gene editing efficiency: 25%). Detailed findings are provided in Fig. 4.

### Experiment 6

#### A targeted mutant mouse was generated through the incubation of sperm cells in the c-TYH medium supplemented with 2 mM MBCD and gRNA-Cas9 plasmid constructs

**Fig. 5:**
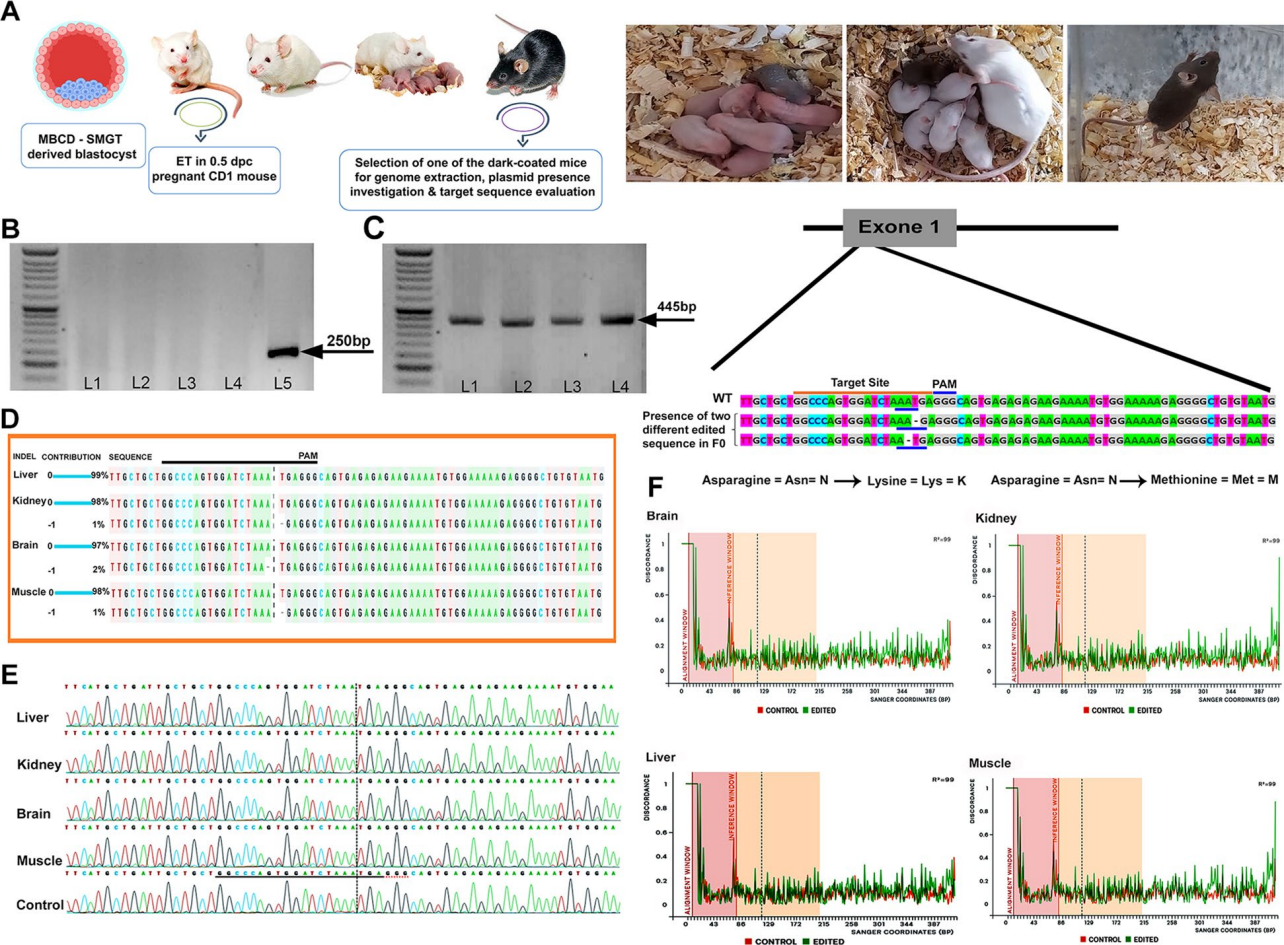
Mosaic genome-edited F0 mouse production. **A** Overview of F0 mouse production: Day 4 blastocysts obtained by incubating sperm in the c-TYH medium with 2 mM MBCD and 20 ng/ µl pgRNA-Cas9 were used for embryo transfer in a 0.5 dpc pregnant mouse. Genome extraction was subsequently performed from a dark-coated F0 mouse to investigate the presence of plasmids and evaluate the target sequence. **B** Detection of gRNA-Cas9 plasmid in the F0 mouse genome: PCR and gel electrophoresis were performed to detect the desired fragment (a 250 bp fragment between the U6 promoter and the guide region), which was not detected in the samples. L1-L4: Kidney, liver, brain, and muscle samples, respectively. L5: Positive control (the pgRNA-Cas9 was used as a template); and L: 50 bp DNA ladder. **C** Gel electrophoresis image of isolated fragments of the gene of interest (*Gdf8*, fragment size: 445 bp) from genomic DNA of the kidney (L1), liver (L2), brain (L3), and muscle (L4) of the F0 mouse. **D**-**F** Direct indel detection using the sequence trace decomposition method through the inference of CRISPR edits (ICE) software. For this detection, Sanger sequencing results of the 445 bp isolated fragments of the mentioned tissues of the F0 mouse were used. **D** The relative contribution of the wild-type and edited sequence (**E**) Sanger sequencing view showing edited sequences in kidney, brain, and muscle tissues, non-edited sequence in liver tissue, and wild-type sequence as the control in the vicinity of the guide region. The guide sequence is represented by the black region that is underlined horizontally. **F** The discordance plot of brain, kidney, liver, and muscle samples of the F0 mouse provides details about the degree of similarity per base between the wild-type (control) and the samples within the specific region surrounding the cut site (inference window)

In order to investigate the presence of plasmid sequence in dark-coated mice derived from ET (F0 founder mice) after repeated cell divisions, one dark-coated mouse was euthanized, and the genome was extracted from the kidney, liver, brain, and muscle. Conventional PCR was performed to isolate the fragment between the U6 promoter and the guide region using the U6-F and antisense strand as forward and reverse primers. However, no plasmid sequence was detected in the samples by electrophoresis on agarose gel (Fig. 5). Next, to study the target site within the *Gdf8* gene, the target fragment was isolated by PCR and sequenced. Finally, computational analysis of chromatograms by ICE showed a targeted cleavage in kidney, brain, and muscle tissues. Two distinct types of genome editing were verified in these three tissues: the same indel in the kidney and muscle and a different indel in the brain. In all the genome-edited samples, a single nucleotide deletion was detected, and the position of the deletion was three and four nucleotides prior to the PAM (-3 and − 4). Therefore, the data showed that the F0 mouse was a mosaic because three distinct sequences (a wild-type sequence and two edited sequences) of the *Gdf8* gene were confirmed in its tissues. Detailed findings are provided in Fig. 5.

## Discussion

The present study demonstrates the efficiency of different concentrations of MBCD in the c-TYH medium in the process of binding and internalizing the CRISPR/Cas9 system into mouse spermatozoa. Additionally, this study determines the transgenic blastocyst production rate achieved by MBCD-SMGT and the efficiency of MBCD-SMGE in producing targeted mutant mouse blastocysts. Finally, our study indicates the potential of the MBCD-SMGT technique for producing targeted mutant mice in a c-TYH medium containing 2 mM MBCD and 20 ng/µl pgRNA-Cas9.

The results of experiment 1 showed that the viability of spermatozoa in the c-TYH medium was lower than in the HTF medium containing 0.4% BSA. Also, at the 2 mM MBCD concentration, the viability, motility, and acrosome integrity were significantly decreased. Similarly, Rahimi et al. used different concentrations of MBCD to transfect rooster spermatozoa; they reported that the viability of transfected sperm in the 0 mM MBCD group was lower compared to the control group. Unlike our findings, in their experiment, the viability of transfected sperm in the 1 mM MBCD group was significantly higher than in the 0 mM group, and there was no significant difference in progressive motility between 0, 1, and 2 mM MBCD groups [29]. Another important finding in our present study (Experiment 2) was the higher levels of extracellular ROS detected in the sperm incubation media in the presence of MBCD compared to the negative control group. It has been demonstrated that MBCD is a BSA substitute [22] that extracts cholesterol from sperm membrane more rapidly and efficiently than BSA, triggering in vitro capacitation [23]. Caliceti et al. further showed that MBCD, unlike BSA, can extract cholesterol from both lipid raft (cholesterol-enriched microdomain) and non-raft fractions [25, 30]. Therefore, MBCD disrupts the lipid raft and influences signaling pathways [24]. Moreover, it has been established that the removal of cholesterol leads to the activation of the NADPH oxidase enzyme found in the lipid raft region. This activation subsequently increases the levels of ROS in both the extracellular and intracellular spaces. Furthermore, ROS have been found to enhance intracellular Ca^2+^ concentration by stimulating calcium channels. The elevation in cytosolic Ca^2+^ concentration also augments NADPH oxidase activity and ROS production [31]. Intriguingly, it has also been observed that with the increase in cytosolic Ca^2+^ concentration, NADPH oxidase assumes a secondary function by serving as a proton channel. This dual role allows for the compensation of charge and pH changes resulting from electron efflux [32]. Consequently, this alteration in pH and the increase in Ca^2+^ concentration activate soluble adenylyl cyclase (sAC) and subsequently trigger the sAC/cAMP/PKA signaling pathway, ultimately leading to capacitation [33].

It has been suggested that the activity of ROS is one of the potential hypotheses regarding the initiation of capacitation events [34]. Moderate levels of ROS have been found to facilitate different physiological processes in spermatozoa, including hyperactivation, capacitation, acrosomal reaction, and zona binding [35]. Moreover, Chen and Resh reported that cholesterol extraction from the plasma membrane by MBCD treatment led to ligand-independent phosphorylation of receptors such as the epidermal growth factor receptor (EGFR). The activation of EGFR initiates the activation of phospholipase C (PLC), which transforms phosphatidylinositol bisphosphate (PIP2) into inositol triphosphate (IP3) and diacylglycerol (DAG). IP3 causes an elevation in intracellular Ca^2+^ concentration, which enhances NADPH oxidase activity and increases the production of ROS. DAG also activates protein kinase C (PKC), which, in turn, triggers the extracellular signal-regulated kinase (ERK) signaling pathway. It has been shown that the ERK signaling pathway participates in sperm capacitation, acrosome reaction, and the phosphorylation of proteins in the axoneme and cytoskeleton. Additionally, this pathway increases protein kinase A (PKA) activity, which is involved in protein tyrosine phosphorylation [36]. It has been revealed that PKA, PKC, and cAMP contribute to an increase in mitochondrial membrane potential and ROS production [34]. In addition, previous studies have demonstrated the activity of the aromatic amino oxidase enzyme in dead sperm, which is considered one factor contributing to increased levels of lipid peroxidation and ROS [37].

As expected, our findings indicate that the extracellular ROS level was higher when sperm were incubated in c-TYH with different MBCD concentrations than HTF with 0.4% BSA. In the present study, at the 2 mM MBCD concentration, the percentage of dead sperm cells significantly increased due to extensive cholesterol extraction from the lipid rafts, high ROS levels, and activation of apoptosis through the inhibition of the PI3K-Akt-Bad signaling pathway [38]. It has been established that ROS, when present in moderate concentrations, activates the PI3K-Akt-Bad signaling pathway, inhibiting apoptosis. However, ROS inhibits this signaling pathway at high concentrations and triggers apoptosis [39]. In our study, an increase in the premature acrosomal reaction in sperm treated with various concentrations of MBCD was seen. Similarly, several studies have indicated that MBCD could induce premature acrosomal reaction [28, 40–42]. Studies have shown that ROS significantly affect calcium homeostasis and protein phosphorylation [43, 44]. For example, Petrov et al. showed that treatment with MBCD increased neurotransmitter exocytosis at the neuromuscular junction. They suggested that the production of ROS following the release of cholesterol increased cytoplasmic Ca^2+^ concentration and synaptic vesicle exocytosis. In their report, the increase in Ca^2+^ concentration is dependent on ROS through the activation of protein phosphatase 2B, causing a rise in the synaptic vesicle exocytosis. Thus, ROS can play a role in regulating proteins involved in exocytosis [31]. The current study also demonstrated that incubation of sperm in the presence of plasmid had no effect on sperm functional parameters, which agrees with the investigations of many groups of researchers [45–51].

The simple and well-known SMGT method, i.e., DMSO-SMGT, was used as a positive control group in the present study. In 2006, Shen et al. used DMSO for in vitro and in vivo transfection of mouse and rabbit sperm cells for the first time. They were able to produce transgenic blastocysts by incubating sperm cells for 10–15 min at 4 °C in the presence of 3% DMSO and 20 ng/ µl pEGFP-N1. In their reports, the GFP-positive blastocyst production rate was 41.7% [19]. Our findings demonstrated that the viability and motility of spermatozoa in the presence of DMSO and cold shock are greatly reduced, which can be attributed to cold shock and the effects of DMSO on the sperm membrane. These findings are in agreement with Kurd et al.’s study, which hypothesized that cold shock causes the joining of environmental ions to the plasma membrane and causes changes in the membrane. They were able to reduce the detrimental effects of cold shock and DMSO on sperm functional parameters through incubation of mouse spermatozoa in an electrolyte-free medium (0.33 M glucose and 4 mg/ml BSA), resulting in improved viability, progressive motility, and an increase in the production rate of transgenic blastocysts obtained from the DMSO-SMGT method [52]. It is well documented that cold shock leads to irreversible suppression of motility and metabolic activities, disruption of the plasma and acrosome membranes, and increased permeability of spermatozoa of different species. In addition, the rapid decrease in environmental temperature and DMSO treatment increases calcium absorption [53, 54]. In our study, treating sperm with DMSO and cold shock increased extracellular ROS and acrosomal reaction like the 2 mM concentration of MBCD.

This study also assessed the copy numbers of exogenous DNA internalized per sperm cell using the absolute real-time PCR technique. Our findings revealed that in the absence of MBCD, approximately 47 copy numbers of exogenous DNA molecules were inserted per sperm cell. This result is in close agreement with the results of Mácha et al. They reported that incubating mouse sperm with 0.5 µg plasmid DNA/5 × 10^6^ sperm cells for 20 min resulted in the entry of 45 copies of plasmid per sperm cell [55]. Furthermore, we demonstrated that the addition of 0.75 mM MBCD to the c-TYH medium caused a significant decrease in the number of exogenous DNA introduced into the sperm when compared to the 0 mM MBCD group (24 vs. 47). Lavitrano et al. reported that postponing of the capacitation reaction in spermatozoa by incubating them in a calcium-free medium can create optimal conditions for the interaction of exogenous DNA and sperm [56]. Several studies have also indicated that the presence of BSA or Ca^2+^ in the sperm incubation medium, which accelerates capacitation, adversely affects exogenous DNA binding [20, 57]. These reports confirm that capacitation reduces exogenous DNA uptake by spermatozoa. We suggest that high extracellular ROS levels may decrease sperm-DNA binding.

In comparison, the level of extracellular ROS in a 0.75 mM concentration of MBCD was significantly higher than the 0 mM concentration of MBCD (5.25-fold increase). There is an expectation of competition between ROS and exogenous DNA to bind to DNA-binding proteins due to their negative charge. In confirmation of our hypothesis, a study showed that the presence of polyanion substances like heparin hinders the interaction between exogenous DNA and spermatozoa [55, 58]. At a 1 mM concentration of MBCD, DNA uptake by sperm was higher than at 0 mM MBCD (88 vs. 47). Although it was expected that sperm-DNA uptake would be reduced in this concentration, similar to 0.75 mM, due to high extracellular ROS levels and low sperm-DNA binding, our results showed an increase in sperm DNA uptake. Though it may be difficult to explain this finding clearly, we hypothesize that at this concentration, the activity of inhibitory proteins that prevent the internalization of bound exogenous DNA to sperm is significantly inhibited and disrupted by changes in membrane cholesterol levels, the activity of microdomains, and the level of protein phosphorylation. It has been demonstrated that only 15–22% of the bound exogenous DNA is transferred to the nucleus of a sperm cell [59, 60], indicating the role of inhibitory mechanisms and proteins in the inhibition of bound DNA transfer to sperm [17]. However, a comprehensive understanding of these mechanisms at the molecular level has not yet been established. Similarly, Oddi and his colleagues quantified the exogenous DNA uptake by boar sperm in the presence of 1 mM MBCD. In their experiment, the incubation of sperm cells in a non-capacitating medium, Tyrode’s basal medium (TBM), caused the entry of 16 copies of exogenous DNA into each sperm cell. However, in the presence of 1 mM MBCD, exogenous DNA uptake increased to 57 copies per sperm cell [28]. The difference in the copy numbers of exogenous DNA uptake by sperm between our study and Oddi’s study is presumably due to various factors. These factors include the difference in the length of the exogenous DNA fragment (10,245 bp vs. 5502 bp), the concentration of exogenous DNA used (20 ng/µl vs. 5 ng/µl), the number of spermatozoa in the incubation medium (2000 sperm/µl versus 100,000 sperm/µl), and variations in binding efficiency among different species. The exogenous DNA-sperm interaction is a reversible ionic phenomenon in which molecules with a negative charge interact with DNA-binding proteins. It has been proven that larger fragments of exogenous DNA are more easily absorbed than smaller fragments due to their higher negative charge [59].

At the 2 mM MBCD concentration, although the inhibitory proteins involved in DNA uptake were presumably lower than at 1 mM MBCD due to the higher cholesterol extraction, there was a significant decrease in the copy numbers of exogenous DNA uptake by sperm. This decrease was attributed to the higher level of extracellular ROS compared to 1 mm MBCD. Consequently, a significant decrease in DNA-sperm interaction and exogenous DNA uptake occurred at the 2 mM MBCD concentration.

In DMSO treatment, the copy numbers of exogenous DNA uptake per sperm cell were 624. It has been shown that DMSO enhances the permeability of the cell membrane to DNA [54]. Notman and his colleagues showed that DMSO reduces molecular transport barriers in the membrane, leading to the formation of membrane pores [61]. Additionally, it has been demonstrated that DMSO acts oppositely to cholesterol in the membrane. While cholesterol makes the membrane compact and cohesive, DMSO causes the membrane to expand and the membrane bilayers to come closer together [54]. Gurtovenko and Anwar also discovered that DMSO, in low concentrations (< 10%), only causes lateral expansion of the membrane and decreases the membrane’s thickness. However, they found that higher concentrations (10–20%) lead to the formation of temporary water pores and a progressive reduction of the membrane bilayers’ thickness [62]. Additionally, de Ménorval and his colleagues demonstrated that DMSO concentrations above 15% increase the membrane’s permeability to Ca^2+^ and increase the concentration of intracellular Ca^2+^ [54]. Recently, Mizuno et al. found that one of the toxic effects of DMSO is the production of ROS. They showed that human umbilical vein endothelial cells were more susceptible to freezing in the presence of DMSO due to higher membrane fluidity and lower antioxidant capacity [63]. Thus, the influence of DMSO on cellular membranes exhibits variability, which can be attributed to several factors, including DMSO concentration, exposure duration, membrane fluidity, and the cell’s resistance to ROS. It seems that the tolerance of sperm against DMSO is low due to their low antioxidant capacity, high sensitivity to ROS, and high fluidity of their cell membranes caused by high levels of polyunsaturated fatty acids [64]. Therefore, it is likely that, unlike other cells, DMSO in a concentration lower than 10% causes the creation of water pores, increases membrane permeability, and leads to an increased entry of exogenous DNA molecules into sperm. Consequently, in this treatment, despite the high level of ROS, there is an increased intake of exogenous DNA due to the creation of pores on the sperm membrane surface and the entry of exogenous DNA through these pores. Hosseini Pajoh et al. conducted a study where they found that treating ram epididymal sperm with 0.1% DMSO at room temperature for 10 min significantly enhanced plasmid uptake compared to incubation with exogenous DNA alone (control group). Their research also showed that 0.1% DMSO did not affect sperm motility, whereas 3% DMSO destroyed spermatozoa. Additionally, their results indicated that plasmid absorption mainly occurred in non-motile spermatozoa during DMSO-SMGT [65]. This finding could potentially explain the lower production rate of transgenic blastocysts observed with the DMSO-SMGT method despite a high number of absorbed copy numbers per sperm cell.

As mentioned earlier, when sperm cells are incubated with MBCD compared to BSA, there is a significant increase in the production of ROS. This elevated ROS production leads to various effects in the treated sperm cells, such as increased intracellular Ca^2+^ concentration, mitochondrial membrane potential, PKA and PKC activity, and ERK signaling pathway activation. These changes ultimately result in hypermotility, a state of increased sperm movement [66]. Numerous studies have highlighted the crucial role of the ERK signaling pathway in flagellar movement and hyperactivated motility. The pathway achieves this by phosphorylating the proteins present in the axoneme, thereby enhancing the movement of the flagellum [67]. Furthermore, it has been demonstrated that MBCD increases the rate of glucose transport into the cell and causes the redistribution of GLUT1 and other intracellular transporter molecules to the plasma membrane [25]. Based on various studies exploring the effects of MBCD on sperm cells, it can be inferred that treating sperm cells with MBCD, as opposed to other methods like DMSO and cold shock, induces hypermotility in live sperm cells. This leads to a notable increase in the number of sperm cells capable of successful interaction. Consequently, utilizing MBCD on sperm cells results in a larger population of transfected motile sperm than DMSO and cold shock.

The current study discovered that a low plasmid copy number uptake by sperm does not necessarily lead to a decrease in the production rate of transgenic embryos. This is due to the fact that sperm act as a factory for cDNA production from mRNA and possess a reverse transcriptase enzyme. Once exogenous DNA interacts with DNA-binding proteins and enters the nucleus facilitated by CD4, transcription occurs. Subsequently, the mRNA is reverse-transcribed by the active reverse transcriptase enzyme in sperm cells, resulting in transcriptionally and translationally active cDNA [6]. Moreover, it has been shown that cellular stress can increase the activity of the reverse transcriptase enzyme [7]. Therefore, it is probable that the concentration of 2 mM MBCD induces a higher activity of the reverse transcriptase enzyme due to oxidative stress. Consequently, the reduced amount of absorbed plasmid DNA is compensated by an increased production of cDNA. In our study, the forward primer used to quantify the copy number uptake by sperm was specifically designed for the U6 promoter sequence. Therefore, the obtained copy number only reflects the initial copy number that entered and does not include the cDNA resulting from endogenous reverse transcriptase activity.

In the present study, no fertilization occurred in the 0 mM MBCD group, which is consistent with the findings of Choi and Toyoda [22]. However, excluding the 0 mM group, there were no significant differences in fertilization rate or early embryonic development among the other treatment groups. This contrasts with Choi and Toyoda’s results, in which they observed a higher fertilization rate at a concentration of 0.75 mM MBCD compared to concentrations of 1 and 2 mM (48% vs. 45% and 3%, respectively) [22]. In our opinion, several factors may have contributed to the low fertilization rate in their study. Firstly, they used denuded oocytes, which can potentially affect the fertilization process. Additionally, they performed IVF in a c-TYH medium without MBCD instead of using an HTF medium containing 0.4% BSA. Lastly, they incubated the sperm cells for 90 min in the presence of different concentrations of MBCD, which might have affected the viability of the sperm. In our study, we observed a significant increase in the production rate of GFP-positive blastocysts when spermatozoa were treated with MBCD compared to using DMSO. Furthermore, there was an upward trend in the rate of GFP-positive blastocyst production with increasing concentrations of MBCD, although this trend was not statistically significant.

One of this study’s objectives was to generate targeted mutant blastocysts and mice using the MBCD-SMGE technique. Blastocysts obtained from the treatment of sperm cells in the c-TYH medium containing 2 mM MBCD, and 20 ng/µl pgRNA-Cas9 were employed for embryo transfer and tracking of indel. By directly applying Sanger sequencing and analyzing the chromatogram of the target sequence within the isolated gene fragment of interest, we observed single nucleotide deletions at the desired site in the blastocyst and mouse born through this technique. Sequencing analysis by ICE software indicated a 25% success rate in targeted mutant embryo production. It has been reported that the efficiency of gRNA-Cas9 or TALENs for indel generation in mammalian embryos ranges from 0.5 to 40.9% per injected zygote, and in mammalian neonates, it ranges from 0 to 41.7% [68].

It is worth noting that in the present study, the confirmed genome-edited blastocyst and mouse displayed a mosaic pattern, i.e., only a subset of cells underwent successful editing, resulting in a mixture of edited and unedited cells. This could be ascribed to the presence of off-target mutations, the absence or low concentration of CRISPR components in the blastomeres resulting from early embryonic divisions, and the timing of editing. Generally, mosaicism occurs when DNA replication precedes CRISPR-mediated genome editing [69]. In the current study, an all-in-one CRISPR platform was used. It has been shown that plasmids are expressed in sperm, but translation occurs between days 0.8–1.3 after fertilization. Thus, translation occurs after the replication stage of the cell cycle in the zygote (S phase) and maybe even after cleavage, resulting in mosaicism [70]. This problem can be solved using the ribonucleoprotein (RNP: Cas9 protein and guide mRNA) system as an alternative to the plasmid system. Treating sperm with RNP provides rapid and efficient action in performing DSB and making indels [71]. In this case, Cas9 nuclease activity and NHEJ occur prior to the S phase of the cell cycle in the zygote, resulting in F0 mice that do not exhibit mosaicism. It has been reported that injection of RNP in conjunction with sperm in the matured oocyte resulted in a single mutant embryo [72].

In this study, the absence of plasmid sequences in the genome extracted from F0 mouse tissues has been demonstrated. This finding suggests that the MBCD-SMGT method may not effectively produce stable transgenic animals, similar to most other SMGT protocols. Therefore, the disadvantage of SMGT, which involves the extrachromosomal arrangement of plasmid DNA, is still observed in the MBCD-SMGT technique. However, it is important to note that the CRISPR/Cas9 system does not require stable expression. The temporary expression of the all-in-one CRISPR/Cas9 platform is sufficient for creating targeted mutations. Thus, this study highlights the potential of combining the MBCD-SMGT technique with CRISPR/Cas9 to generate targeted mutant blastocysts and mice. Furthermore, it is worth noting that, similar to other studies utilizing the CRISPR/Cas9 system, concerns regarding its off-target effects persist. However, these concerns can be addressed through the use of precise tools in designing the guide sequence, Cas9 nickase enzyme, and RNP platform.

## Conclusion

In conclusion, this report represents the first successful production of targeted mutant mice using the incubation of sperm cells in a c-TYH medium with 2 mM MBCD and 20 ng/ µl gRNA-Cas9 plasmid construct. Our findings strongly support the concept that cholesterol removal from the sperm membrane by MBCD enhances extracellular ROS production by spermatozoa. Additionally, these negatively charged molecules compete with exogenous DNA molecules for binding to the DNA-binding receptor. Furthermore, our observations indicate that the use of MBCD leads to a larger population of transfected motile sperm and a higher production rate of GFP-positive blastocysts compared to the utilization of DMSO and cold shock methods. However, it is crucial to mention that our study also revealed the presence of the mosaicism phenomenon. To overcome this challenge, we propose using the ribonucleoprotein system as an alternative to the all-in-one plasmid system. Overall, this study highlights the significant potential of the MBCD-SMGE technique for generating targeted mutant mice. This technique holds enormous promise for modeling human diseases and improving desirable traits in animals.

## Data Availability

All the necessary data are present in this manuscript. Any additional information required to reanalyze the data reported in this paper is available from the corresponding author upon request.

## Abbreviations

CRISPR/Cas9: Clustered regularly interspaced short palindromic repeats (CRISPR)/CRISPR-associated protein
MBCD-SMGT: Methyl β-cyclodextrin-sperm-mediated gene transfer
ROS: Reactive oxygen species
c-TYH: Choi- Toyoda Yokoyama Hosi
pgRNA-Cas9: pCAG-eCas9-GFP-U6-gRNA
IVF: In vitro fertilization
MBCD-SMGE: Methyl β-cyclodextrin -sperm-mediated gene editing
GFP: Green fluorescent protein
Indel: Small insertion or deletion mutations
gRNA: Guide RNA
PAM: Protospacer adjacent motif
bp: Base pair
DSB: Double-strand break
NHEJ: Nonhomologous end joining
HDR: Homology-directed repair
ICSI: Intra cytoplasmic sperm injection
βCDs: β-cyclodextrins
PVA: Poly vinyl alcohol
BSA: Bovine serum albumin
HTF: Human tubal fluid
mKSOM: Modified potassium simplex optimization medium
FHM: Flushing holding medium
UTM: Uterine transfer medium
pCAG-GFP: plenti-CAG-gate-FLAG-IRES-GFP
LB: Luria-Bertani
sgRNA: Single-guide RNA
*Gdf8*: Growth differentiation factor-8
PMSG: Pregnant mare serum gonadotropin
hCG: Human chorionic gonadotropin
COCs: Cumulus-oocyte complexes
PBS: Phosphate-buffered saline
LDCA: Luminol-dependent chemiluminescence assay
RLU: Relative Light Units
*Actb*: β-actin gene
2PN: Two pronuclei
IVC: In vitro culture
ICE: Inference of CRISPR edits
ET: Embryo transfer
VP: Vaginal plug
SEM: Standard error of the mean
ANOVA: Analysis of variance
sAC: Soluble adenylyl cyclase
EGFR: Epidermal growth factor receptor
PLC: Phospholipase C
PIP2: Phosphatidylinositol bisphosphate
IP3: Inositol triphosphate
DAG: Diacylglycerol
PKC: Protein kinase C
ERK: Extracellular signal-regulated kinase
PKA: Protein kinase A
RNP: Ribonucleoprotein
