# Correction: Methyl β-Cyclodextrin-sperm-mediated gene editing (MBCD-SMGE): a simple and efficient method for targeted mutant mouse production

**DOI:** 10.1186/s12575-024-00231-8

**Published:** 2024-02-16

**Authors:** Parisa Moradbeigi, Sara Hosseini, Mohammad Salehi, Asghar Mogheiseh

**Affiliations:** 1 Department of Clinical Sciences, School of Veterinary Medicine, Shiraz University, P. O. Box: 7144169155, Shiraz, Iran; 2 Cellular and Molecular Biology Research Center, Shahid Beheshti University of Medical Sciences, P.O. Box: 193954717, Tehran, Iran; 3 Hasti Noavaran Gene Royan Co, Tehran, Iran; 4 Department of Biotechnology, School of Advanced Technologies in Medicine, Shahid Beheshti University of Medical Sciences, Tehran, Iran

**Correction: Biol Proced Online 26, 3 (2024)**

10.1186/s12575-024-00230-9

Following publication of the original article [1], the author reported two errors:

1. The error in Fig. 3 displaying blastocysts obtained from the 2mM CAG-GFP group instead of the intended 2mM gRNA-Cas9 group, as clarified in the figure legend within the article. The correct figure is shown in this article. Also, in the figure legend, the word 'SMGT' has been appropriately changed to 'SMGE' to reflect the accurate terminology.
2. The repetition of the title ‘argeted indel was validated in blastocysts derived from sperm incubated with 2 mM MBCD and 20 ng/μl gRNA-Cas9 plasmid constructs’ under Experiment 5 in the results section.

**Fig. 3:**
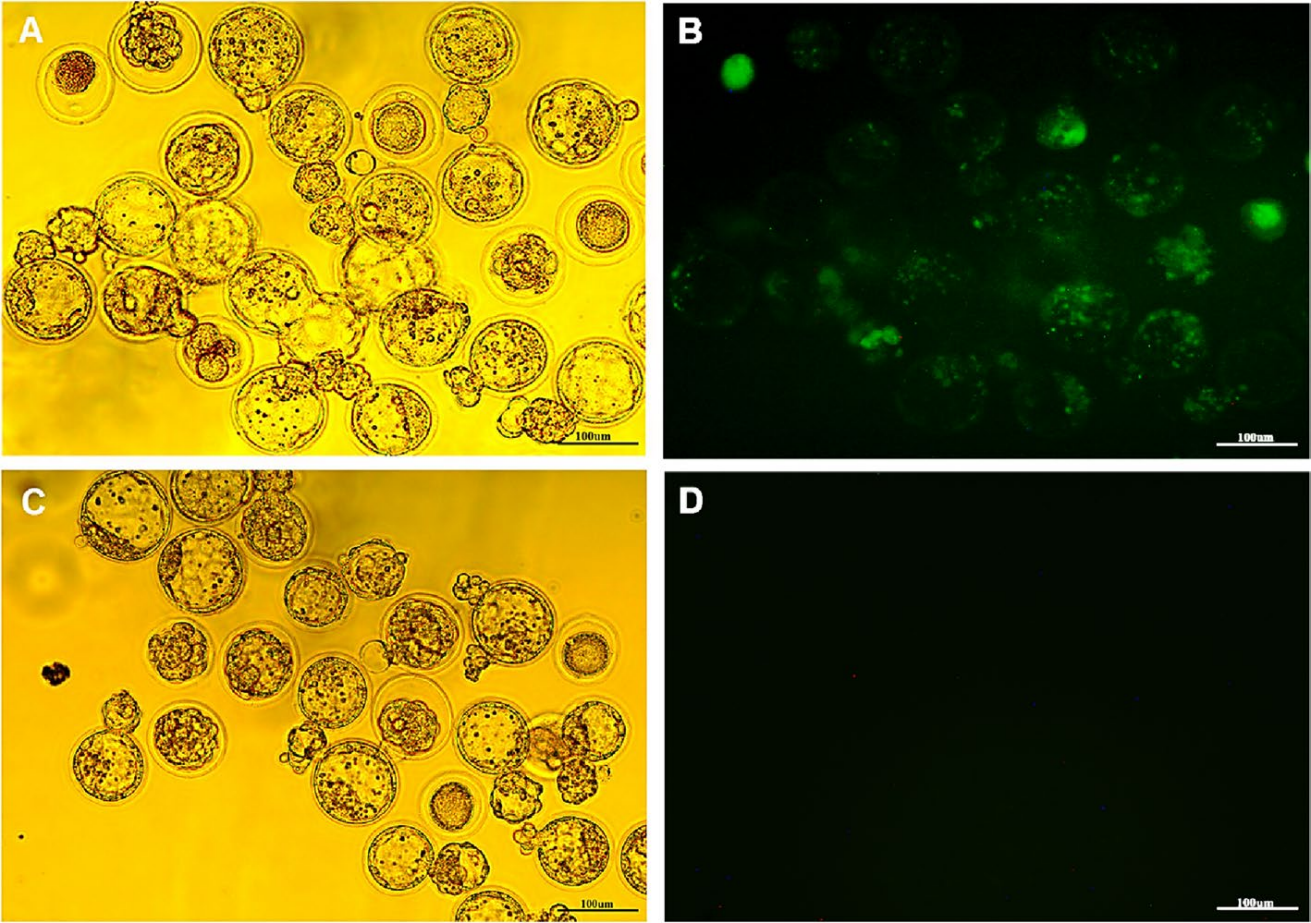
Murine GFP-positive blastocyst production using the MBCD-SMGE and IVF-IVC methods. **A** Bright field and (**B**) fluorescent field images of day 4 mouse blastocysts obtained from the MBCD-SMGE and conventional IVF-IVC methods. In the MBCD-SMGE method, sperm were incubated in the c-TYH medium supplemented with 2 mM MBCD and 20 ng/μl pgRNA-Cas9. **C** Bright field and (**D**) fluorescent field images of day 4 mouse negative control blastocysts. Scale bar size: 100 μm

The original article has been corrected.
